# Development and Characterization of Citalopram-Loaded Thermosensitive Polymeric Micelles for Nasal Administration

**DOI:** 10.3390/pharmaceutics17091147

**Published:** 2025-09-01

**Authors:** Fatima Rajab, Bence Sipos, Gábor Katona, Ildikó Csóka

**Affiliations:** Institute of Pharmaceutical Technology and Regulatory Affairs, University of Szeged, Eötvös Street 6, H-6720 Szeged, Hungary; fatima.rajab@szte.hu (F.R.); katona.gabor@szte.hu (G.K.); csoka.ildiko@szte.hu (I.C.)

**Keywords:** intranasal, polymeric micelle, Pluronic F127, Poloxamer 188, thermosensitive, citalopram, nanocarriers

## Abstract

**Background/Objectives:** The intranasal (IN) route of administration is a promising non-invasive approach for brain targeting, bypassing the blood–brain barrier and enhancing bioavailability. Citalopram hydrobromide (CT), a widely prescribed sparingly water-soluble selective serotonin reuptake inhibitor (SSRI), faces challenges with oral and intravenous administration, including delayed onset, adverse effects, and patient compliance issues. **Methods:** This study aimed to develop a novel thermoresponsive polymeric micelle (PM) system based on Pluronic^®^ copolymers (Pluronic F127 and Poloxamer 188) improving CT’s solubility, stability, and nasal permeability for enhanced antidepressant efficacy. A preliminary study was conducted to select the optimized formulation. The preparation process involved using the thin-film hydration method, followed by freeze-drying. Comprehensive evaluations of optimized formulation characteristics included Z-average, polydispersity index (PdI), thermal behavior (lower critical solution temperature, LCST), encapsulation efficiency, X-ray powder diffraction (XRPD), thermodynamic solubility, and biological stability. Additionally, in vitro CT release and CT permeability in nasal conditions were studied. Stability under storage was also evaluated. **Results:** The optimized CT-PM formulation showed nanoscale micelle size (Z-average of 31.41 ± 0.99 nm), narrow size distribution (polydispersity index = 0.241), and a suitable thermal behavior for intranasal delivery (lower critical solution temperature (LCST) ~31 °C). Encapsulation efficiency reached approximately 90%, with an amorphous structure confirmed via XRPD, leading to a 95-fold increase in CT solubility. The formulation demonstrated appropriate biological and physical stability. In vitro studies showed a 25-fold faster CT release from optimized formulation compared to the initial CT, while CT-PM permeability in nasal conditions increased four-fold. **Conclusions:** This novel nanoscale thermosensitive formulation is a value-added strategy for nasal drug delivery systems, offering enhanced drug solubility, rapid drug release, stability, and improved permeability. This smart nanosystem represents a promising platform to overcome the limitations of conventional CT administration, improving therapeutic outcomes and patient compliance in depression management.

## 1. Introduction

Depression is one of the most common mental health diseases and a dominant cause of disability around the world. According to the World Health Organization (WHO), it impacts approximately 280 million people worldwide and is associated with 700,000 suicides annually [1]. Many obstacles result from depression that could decrease the patient’s quality of life, such as difficulties in concentration, lack of social activities, and suicidal thoughts [2]. Moreover, it has been recognized as an individual risk factor for cardiovascular disease [3,4]. Although several treatment approaches, including psychotherapies and pharmaceutical treatments, are applied, under 50% of patients fully recover [5,6]. Selective serotonin reuptake inhibitors (SSRIs), the current first line, are widely used antidepressants due to their tolerability, efficacy, and safety [7]. Citalopram hydrobromide (CT) is one of the most prescribed SSRIs, which has the safest drug interaction profile due to its relatively low protein binding compared to other SSRIs [7]. It also has the highest selectivity for inhibiting serotonin reuptake compared to noradrenaline reuptake [7,8]. CT has been used as an efficient treatment in anxiety disorders such as panic disorder and obsessive-compulsive disorder, as well as post-stroke depression, dementia, depression, and addiction disorder [8,9,10,11,12,13]. Currently, the available CT formulations in the market are oral products, i.e., tablets and intravenous (IV) injections. Orally administered CT faces significant challenges such as the delayed onset of action and gastrointestinal disorders, including nausea, vomiting, diarrhea, appetite change, and constipation, while IV injections, despite their faster onset, are undesirable due to their invasive delivery method [14]. These issues negatively impact on the patient’s life quality and compliance, which are crucial for the success of therapy [15].

Intranasal (IN) administration offers a promising non-invasive approach. The unique anatomy of the nasal cavity and its direct connection with the brain through the olfactory and trigeminal pathways allow for bypassing of the blood–brain barrier (BBB) and offer targeted nose-to-brain drug access, which is especially beneficial in acute situations [16]. Additionally, the high vascularity and large surface area of the nasal mucosa enhance systemic drug absorption, allowing for the use of lower drug doses compared to oral administration with enhanced efficiency [17]. These outstanding advantages of IN delivery alongside the avoidance of the hepatic first-pass metabolism play a remarkable role in accelerating the drug onset of action and lowers the risk of systemic side effects [17]. CT was employed as a model active agent in order to enhance its bioavailability, as it is considered suitable for intranasal administration due to its lipophilicity and preferred molecular weight (405.31 g/mol) below 1000 g/mol [18].

The promising potential of intranasal delivery of antidepressant-loaded nanosystems was reported in previous studies [19,20,21]. Venlafaxine was loaded into IN PLGA nanoparticles, which demonstrated effective brain uptake with low cytotoxicity and an enhanced in vivo antidepressant-like effect [19]. Duloxetine has also been incorporated into an IN thermoreversible cubosomal gel containing lipids, PF127, and P188. It showed significant greater brain bioavailability [20]. A nanoemulsion was designed for the IN delivery of paroxetine and exhibited a remarkable increase in in vivo permeability [21].

Polymeric micelles (PMs) are nanosized self-assembled colloidal structures created from amphiphilic biocompatible copolymers. These co-polymers, formed of hydrophilic head groups and hydrophobic tail groups, self-arrange into core–lipid micelles in aqueous media. This unique structure can ameliorate the solubility and bioavailability of many water-insoluble drugs [22,23]. Furthermore, its biological stability protects the drug from enzymatic degradation, while its small size (10–100 nm) allows for enhanced permeability and expedites infiltration through the brain’s narrow capillaries [24,25]. Various co-polymers, including thermosensitive polymers, can form PMs under the title of smart drug delivery systems [26,27]. These polymers, such as Pluronics^®^, are fully water soluble and exist in an extended form below a specific temperature, the lower critical solution temperature (LCST), which is considered their phase transition temperature. When the medium temperature is raised over the LCST, the micelle undergoes a size reduction due to the dehydration process of the polymer’s hydrophilic groups, which decreases their solubility and leads to a shrinkage in micelle size [28]. It was also shown that Pluronics^®^ modulate the efflux transmembrane transporter P-glycoprotein at the BBB, thus boosting the brain penetration of Pluronic^®^-loaded antidepressants [16]. Previous research has demonstrated the advantages of intranasal Risperidone-loaded Pluronic micelles (Pluronic^®^ F127 and Pluronic^®^ F108) in providing rapid drug release with higher permeation rate [29]. Pluronic^®^ F127 (PF127) (also known as Poloxamer^®^ 407) is characterized by its thermoresponsive properties at intranasal temperature (32–35 °C), along with its ability to form stable micelles and encapsulate hydrophobic drugs, thereby augmenting their solubility and stability in aqueous environments, with a hydrophilic–lipophilic balance (HLB) value of 22 [30,31,32]. In contrast, Poloxamer^®^ 188 (P188) (also known as Pluronic F68) exhibits weaker thermosensitive behavior but offers higher solubilization ability due to its greater hydrophilicity and higher HLB value (29) [32,33,34]. It was also reported that P188 shows a “stealth” effect by minimizing protein adsorption and avoiding rapid elimination by the reticuloendothelial system (RES) [32]. These two Pluronics^®^ have received Food and Drug Administration (FDA) approval for use in pharmaceutical and biomedical applications in vivo and were recognized as biocompatible and safe for intranasal delivery with the absence of mucosal irritation or toxicity [32,35,36]. The combination of PF127 with P188 in intranasal delivery is expected to achieve balanced thermal behavior, boosted drug solubility and permeability, faster onset of action, enhanced bioavailability, and optimized drug encapsulation, leading collectively to superior brain penetration while also addressing the challenge many patients face with swallowing oral dosage forms and avoiding the inconvenience of IV administration.

Eventually, the present study describes a novel formulation of optimized Pluronic-based thermoresponsive micelles of citalopram for intranasal administration. Based on preliminary studies, a carrier system was developed with adequate colloidal properties, which could overcome the limitations of systemic side effects and retarded onset of action by enhancing citalopram solubility and exploiting the temperature-triggered drug release properties of thermosensitive PMs highlighting the critical role of optimizing thermoresponsive polymer blends to improve the intranasal delivery of antidepressants. This manuscript details the comprehensive physicochemical characterization, in vitro release, and in vitro evaluation of drug permeability in intranasal conditions, laying the groundwork for future advancements in the targeted intranasal delivery of antidepressants with appropriate therapeutic efficacy.

## 2. Materials and Methods

### 2.1. Materials

Pluronic^®^ F127 (PF127) (PEG_95_-PPG_62_-PEG_95_ (poly(ethylene-glycol)–block–poly(propylene-glycol)–block–poly (ethylene glycol); average molecular weight: 12,500 Da) was acquired from Sigma Aldrich Co., Ltd. (Budapest, Hungary). Poloxamer^®^ 188 (P188) (PEG_38_-PPG_29_-PEG_38_(poly(ethylene-glycol)–block–poly(propylene-glycol)–block–poly (ethylene glycol); average molecular weight: 8600 Da) was acquired from ThermoFisher GmbH (Kandel, Germany). Citalopram hydrobromide (CT; 1-[3-(dimethylamino) propyl]-1-(4-fluorophenyl)-1,3-dihydro-2-benzofuran-5-carbonitrile hydrobromide; water solubility: <0.1 mg/mL; logP: 3.58; molecular weight: 405.31 g/mol; oral bioavailability: 80%) was purchased from Tokyo chemical industry co., LTD (Tokyo, Japan). Ethanol 96% *v*/*v* was acquired from Merck, Ltd. (Budapest, Hungary). NaCl 0.9% (*w*/*v*) was purchased from B. Braun Melsungen AG (Melsungen, Germany). The simulated nasal electrolyte solution (SNES pH 5.6) was prepared using the following materials: 0.59 g/L of anhydrous calcium chloride (CaCl_2_), 8.77 g/L of sodium chloride (NaCl), and 2.98 g/L of potassium chloride (KCl) dissolved in 1000 mL of purified water and adjusted to pH 5.6 [37]. The phosphate buffer solution (PBS pH 7.4) contains 0.20 g/L KCl, 8.00 g/L NaCl, 0.12 g/L KH_2_PO_4_, and 1.44 g/L Na_2_HPO_4_ × 2 H_2_O dissolved in 1000 mL purified water and adjusted to pH 7.4 [38]. The artificially simulated cerebrospinal fluid (aCSF) was prepared from 0.5 mM Na_2_SO_4_, 5.8 mM D-glucose, 0.5 mM KH_2_PO_4_, 0.2 M NaCl, 1.2 mM CaCl_2_, 0.5 mM KH_2_PO_4_, 1.8 mM MgCl_2_, and 2 mM KCl and set to pH 7.4 [39]. All the chemicals of the dissolution media—SNES, PBS, and CSF—were obtained from Sigma-Aldrich Co., Ltd. (Budapest, Hungary). Analytical-grade acetonitrile was obtained from Merck Ltd. (Budapest, Hungary). The purified water in all experiments was obtained from a Milli-Q^®^ Gradient Water Purification System (Merck Ltd., Budapest, Hungary).

### 2.2. Quantitative Analysis of CT via High-Performance Liquid Chromatography (HPLC)

The quantitative determination of CT was performed via high-pressure liquid chromatography (HPLC) using an Agilent 1260 Infinity (Agilent Technologies, Santa Clara, CA, USA) instrument. The stationary phase was a Kinetex^®^ C18 column (5 µm, 150 mm × 4.6 mm, Phenomenex, Torrance, CA, USA). The mobile phases were the following: (A) 0.1 M sodium acetate buffer (pH 7.0) and (B) methanol. The separation was performed by gradient elution with the following program: The initial (A:B) ratio of 45:55 remained until 1 min, which changed to a ratio of 30:70 by 5 min, which remained until 7 min. The injection volume was 10 µL. The separation was performed for 7 min at 30 °C with a flow rate of 0.8 mL/min. Chromatograms were detected at 239 ± 4 nm using a UV-Vis diode array detector. The retention time was 7.57 min. The limit of detection (LOD) and limit of quantification (LOQ) of CT were 2.49 and 7.55 ppm, respectively. The HPLC method was validated based on robustness and repeatability. The calibration was performed from 2 to 10 µg/mL and from 0.2 to 1.0 mg/mL, where the determination coefficients of linearity (R2) values were 0.9996 and 0.9992, respectively. Chromatograms were evaluated using ChemStation B.04.03 Software (Agilent Technologies, Santa Clara, CA, USA).

### 2.3. Formulation of CT-Loaded Polymeric Micelles

**Figure 1:**
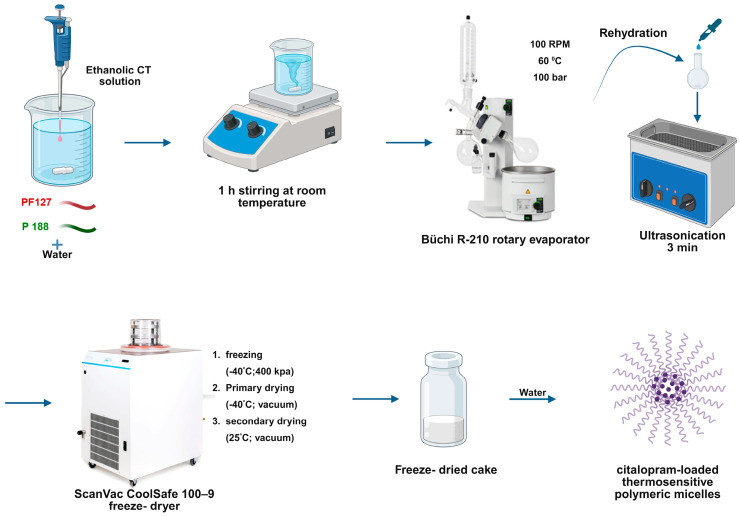
Schematic presentation of the formulation of citalopram-loaded thermosensitive polymeric micelles.

The thin-film hydration method was applied to formulate CT-PMs. Various amounts of PF127, as presented in Table 1, and 40 mg of P188 were dissolved in a mixture of 4 mL of water and 16 mL of 96% ethanol. Then, 2 mL of 5 mg/mL CT ethanolic solution was added. This mixture was stirred constantly at room temperature for one hour, then transferred to a round-bottom flask for drying via a Büchi R-210 (Büchi, Flawil, Switzerland) rotary vacuum evaporator at 60 °C for approximately one hour. The pressure gradually reduced from 1000 to 100 mbar at a rate of 100 mbar per minute. The generated thin film was rehydrated with 10 mL of purified water and ultrasonicated at room temperature for 3 min. After rehydration, the formulations were fractioned into 1 mL samples in vials and freeze-dried using a ScanVac CoolSafe 100–9 (LaboGene, ApS, Lynge, Denmark) at −40 °C under a 0.013 mbar pressure for 12 h. The secondary drying was for 6 h under a 0.013 mbar pressure at 25 °C. The preparation method is presented in Figure 1.

### 2.4. Characterization of the Polymeric Micelles

#### 2.4.1. Determination of LCST

The dynamic light scattering (DLS) method was employed to monitor variations in the Z-average, which refers to the micelle size, and polydispersity index (PdI), as the micelle size distribution, of the CT-loaded PM to determine the LCST via Malvern Zetasizer Nano ZS (Malvern Instruments, Worcestershire, UK). The freeze-dried samples, prepared using ScanVac CoolSafe 100–9 (LaboGene, ApS, Lynge, Denmark), by freezing and drying at −40 °C under a 0.013 mbar pressure for 12 h, followed by secondary drying for 6 h under a 0.013 mbar pressure at 25 °C, were dissolved in purified water. Then, the samples were transferred to disposable folded capillary cells and kept inside during the process of measuring at increasing temperatures, ranging from 25.0 to 40 °C at 1 °C increments, with a refractive index of 1.6 [29,40,41]. The temperature at which a sharp increase in Z-average is observed, followed by a radical descent, is considered the LCST [29]. All measurements were carried out in triplicate (*n* = 3), and the results are expressed as the mean ± SD.

#### 2.4.2. Determination of Micelle Size and Size Distribution

The investigations into the Z-average and PdI of the optimized formulation and blank micelles were carried out by the DLS method (Malvern Instruments, Worcestershire, UK) at an approximate temperature of 35 °C to closely mimic the nasal cavity temperature and assess micelle thermal behavior [31]. All measurements were carried out in triplicate (*n* = 3), and the results are expressed as the mean ± SD.

#### 2.4.3. Determination of Encapsulation Efficiency (EE%)

The indirect method was used to determine the encapsulation efficiency of CT-PM [42]. The freeze-dried samples were dissolved in 1 mL of purified water and placed in Eppendorf^®^ tubes. The Hermle Z323 K high-performance refrigerated centrifuge (Hermle AG, Gosheim, Germany) was used to separate the CT-loaded micelles from the aqueous media by centrifugation with a speed of 13,500 rpm at 4 °C for 45 min. Quantitative measurements were performed via HPLC. The measurements were conducted in triplicate (*n* = 3) and the results are expressed as the average ± SD. The following equation was applied to calculate EE:

(1) $$EE\;\left(\%\right)=\;\frac{Initial\;CT\;\left(\mathrm{mg}\right)-Measured\;CT\;in\;the\;suprenatant\;\;(\mathrm{mg})}{Initial\;CT\;(\mathrm{mg})}\;\times 100$$

#### 2.4.4. Determination of the Thermodynamic Solubility

The saturation method was utilized to determine the enhancement of CT’s thermodynamic solubility in water [42]. An excessive amount of freeze-dried cakes was added to 1 mL of dissolution medium at 25 °C until visible sedimentation occurred, and continuously stirred for 72 h. The solutions were filtered through a 0.22 µm polyether sulfone (PES) membrane filter. The CT concentration in the filtrate was determined via HPLC. All measurements were performed in triplicate (*n* = 3), and the results are expressed as the mean ± SD.

#### 2.4.5. Biological Stability Test

The effect of the presence of our optimized formulation in biological media SNES (pH 5.6), PBS (pH 7.4) and CSF (pH 7.4) on micelle size and zeta potential was evaluated using DLS. The freeze-dried samples were dissolved in the biological media. The solutions were incubated at 35 °C for SNES and 36.5 °C for PBS and CSF [43,44]. Then, aliquots were taken at pre-established time intervals (0, 0.5, 1, and 2 h). The measurements were performed in triplicate (*n* = 3), and the results are presented as means ± SD.

### 2.5. Physical Stability

Physical stability was tested to describe the colloidal stability of the polymeric micelles upon storage. Samples were investigated in the liquid and solid states via DLS. Samples were investigated under two conditions, based on the ICH Q1A (R2) guideline [45]: 5 ± 3 °C in a refrigerator and at 25 ± 2 °C under ambient conditions. Solid samples were dissolved in purified water at each pre-determined time point. The measurements were performed in triplicate (*n* = 3), and the results are presented as means ± SD.

### 2.6. X-Ray Powder Diffraction Study

The crystalline structure of the freeze-dried products was characterized with X-ray powder diffraction (XRPD) using a Bruker D8 Advance X-ray diffractometer (Bruker AXS GmbH, Karlsruhe, Germany) with Cu K λI radiation (λ = 1.5406 Å) and a VANTEC-1 detector. In total, 40 kV of voltage and 40 mA of amperage were used during the measurements. The angular range was 3° to 4° 2θ with a step time of 0.1 s and a step size of 0.007°. The manipulations and evaluations were carried out using EVA Software v6.

### 2.7. In Vitro Nasal Applicability Studies

#### 2.7.1. In Vitro Drug Release Study

The dialysis bag method was applied to perform the drug release study under sink conditions in case of polymeric micelle formulation. Initially, 1 mL of the in-water dissolved freeze-dried formulation (concentration: 1 mg/mL) and initial CT aqueous suspension (the reference, concentration: 1 mg/mL) was placed in presoaked dialysis tubes (Spectra/Por^®^, MWCO 12–14 kDa, Spectrum Laboratories Inc., Rancho Dominguez, CA, USA) and fully submerged in 25 mL of SNES (pH 5.6) at 35 ± 0.5 °C to be stirred at 100 rpm for 1 h using a Hanson SR8 Plus apparatus (Teledyne Hanson Research, Chatsworth, CA, USA). Sample volumes (100 µL) were withdrawn at 1, 3, 5, 10, 15, 30, and 60 min. HPLC was employed to measure the quantity of dissolved CT in the dissolution medium. The measurements were carried out in triplicate (*n* = 3) and the results are presented as means ± SD.

#### 2.7.2. Mathematical Analysis of the Drug Release

The obtained cumulative CT release data of the optimized formulation and CT aqueous suspension were subjected to different mathematical approaches (zero-order, first-order, Hixson–Crowell, Higuchi, and Korsmeyer–Peppas models). The DDsolver^®^ add-in software was applied to interpret the release kinetics and determine the rate constant (K) and regression coefficient (R^2^) values of each model. The optimal model was chosen based on the highest value of R^2^ [46,47].

#### 2.7.3. In Vitro Drug Permeation Study

In order to investigate the formulation tendencies to passive diffusion as the main absorption mechanism at the nasal mucosa, a modified Side-by-side^®^ horizontal diffusion test was performed. An isopropyl myristate-impregnated cellulose membrane with a surface of 0.785 cm^2^ was utilized as the diffusion barrier between the donor and acceptor cells. The study was conducted at 36.5 °C. The volume of both cells was 9 mL. The donor medium was composed of SNES (pH 5.6) while the acceptor phase was PBS (pH 7.4). Then, 50 µL samples were withdrawn from the acceptor phase in predetermined time intervals, and the quantification of CT was performed via HPLC. The Flux (J) (µg/cm^2^) was quantified by dividing the amount of CT permeated through the membrane by the surface of the inserted membrane and the experiment’s duration (µg/cm^2^/h). The permeability coefficient (K_p_) was calculated from J and the drug concentration in the donor phase (Cd (µg/cm^3^)) according to the following equation:

(2) $$K_{p}\left[\frac{\mathrm{cm}}{h}\right]=\frac{J}{C_{d}}$$

### 2.8. Statistical Analysis

Statistical analyses were performed using GraphPad Prism version 10.1.2 (GraphPad Software, San Diego, CA, USA). One-way analysis of variance (ANOVA) was applied, with *p*-values less than 0.05 considered statistically significant. Data are presented as the mean ± standard deviation (SD) based on three independent experiments (*n* = 3).

## 3. Results

### 3.1. Characterization of the Micelles in a Liquid State

#### 3.1.1. Preliminary Studies

The optimal formulation was selected based on the critical parameters of thermosensitive polymeric micelles. Z-average is an essential attribute as it affects pharmacokinetics, stability, and the capability of brain penetration [48]. The optimal range of Z-average is 10–100 nm [24,49]. PdI is also a required parameter due to its indication to the uniformity degree of the micelle size. For polymeric micelles, PdI desirable value is ≤0.3 as it ensures higher stability and less aggregation tend [49,50]. Evaluation Thermal response, which is presented as LCST, is considered a key-factor in thermoresponsive polymeric micelles. LCST value should be high enough to prevent triggered drug release at ambient temperature, ensuring stability and facilitating the administration, and low enough to induce drug release at intranasal temperature (32–35 °C) [32]. Z-average, PdI, and LCST were measured by DLS method. According to the results presented in Table 2, CT-PM3 exhibited the most favorable characteristics. In contrast, formulations CT-PM1 and CT-PM2 showed higher PdI values, suggesting less uniform micelle distribution, while CT-PM4 exhibited a significantly larger particle size (90.13 ± 0.23 nm) and a higher LCST (36 °C), which may reduce its suitability for intranasal administration.

Based on these findings, CT-PM3 was selected as the optimal formulation for further development and evaluation.

#### 3.1.2. Determination of the LCST

**Figure 2:**
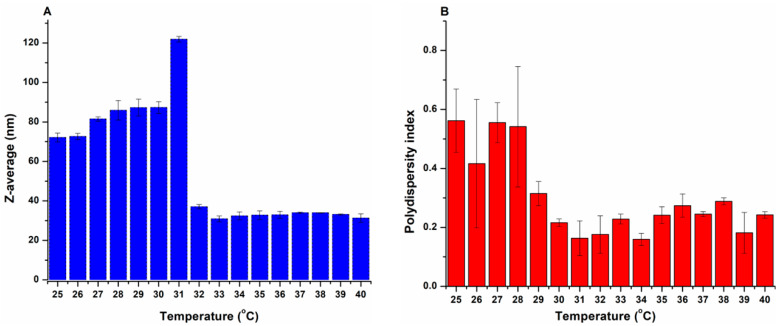
Variations in micelle size (expressed as Z-average) (**A**) and variations in micelle size distribution (expressed as polydispersity index) (**B**) within the temperature range of 25–40 °C. Results are presented as means ± SD (*n* = 3).

In order to assess the thermal behavior of CT-loaded polymeric micelles, the Z-average and PdI of the optimized formulation (CT-PM3) were measured at a temperature range of 25 to 40 °C. As shown in Figure 2, the Z-average showed higher values at temperatures under 32 °C with PdI > 0.3, which indicates a polydisperse size distribution. A sharp increase at approximately 31 °C was followed by a monodisperse fall (PdI ≤ 0.3) in Z-average. This remarkable situation suggests an ideal LCST value of 31 °C for our optimized formulation, as it exceeds room temperature yet remains below the nasal temperature (35 °C). This polydispersity increase in size at lower temperatures might be connected to the formation of large, loose clusters of correlated micelles and the existence of some monomers. At higher temperatures, these aggregations separate as a result of the dehydration and degradation of PEG chains. Meanwhile, hydrophobic groups in the micelle’s core intensify, the hydrogen bonds (H-bonds) of the hydrophilic groups in the micelle’s corona with the aqueous solution weaken, and the monomers vanish and eventually lead to a monodisperse reduction in micelle size [51,52,53,54].

#### 3.1.3. Micelle Size and Size Distribution

The DLS method was used to measure the Z-average and PdI of our optimized formulation and blank micelles at 35 °C. Nose-to-brain delivery could ideally be achieved with a particle size below 200 nm. Furthermore, the optimal size range of polymeric micelles is from 10 to 100 nm, which optimizes circulation time and evades the uptake by monophagocytic system (MPS) and renal filtration [24,49]. As shown in Table 3, the Z-average and PdI of CT-PM3 were within the acceptable range. Additionally, the size of the CT-loaded PM showed significantly increased Z-average compared to the blank PM (*p* < 0.0001). Probably, the added drug particles interacted with the polymer’s molecules, which led to a reduction in the number of surfactant molecules per micelle compared to the pure Pluronic^®^ solution. Additionally, the concentration of Pluronic^®^ F127 (1.1% *w*/*v*) and Poloxamer^®^ 188 (0.4% *w*/*v*) in our optimized formulation is above their critical micelle concentration (CMC), approximately 0.1 and 0.03%, respectively. As a result, the extra molecules are likely involved in the formation of additional micelles, which leads to a reduction in Z-average [55,56]. Moreover, the intermolecular interactions could be formed between the CT molecules and the hydrophobic core of micelles via Van der Waals bonds, resulting in a more compact micelle core compared to unloaded micelles [57].

The narrow size distribution is also considered crucial for PM to ensure high stability, a predictable drug release profile, and consistent cellular uptake [58]. PdI is a criterion to evaluate the width of the size distribution of particles in a range of 0 to 1. The PdI value ≤ 0.3 signifies a monodisperse size distribution, which is highly desirable [49,50]. The results of PdI of both blank and CT-loaded micelles were less than 0.3, which indicates a homogenous particle size distribution.

The zeta potential of our formulation is almost negligible, yet the charge of both used co-polymers is near-neutral. It has been reported in previous studies that micelles with neutral or mildly negative surface charge can efficiently cross the nasal mucosa and exhibit prolonged blood circulation [29,59].

#### 3.1.4. Characterization of Solubilization

The thermodynamic solubility and the encapsulation efficiency of the optimized formulation were determined. Using the saturation method, the thermodynamic solubility of CT revealed a 95-fold increase (*p* < 0.0001), from 15.460 ± 0.032 µg/mL to 1479.342 ± 0.145 µg/mL, upon incorporation into polymeric micelles (CT-PM). The highly achieved encapsulation efficiency of CT, reaching 88.85 ± 1.23%, could explain this CT solubility enhancement. The lipophilic drug incorporation into the micelle core occurs by forming hydrophobic interactions, such as Van der Waals bonds, with PPO groups in the micelle core, while the hydrophilic shell facilitates solubilization by forming H-bonds with the aqueous environment [23,60]. Moreover, the nanoscale dimensions of mixed micelles play an important role in augmenting solubility [23,61].

#### 3.1.5. Biological Stability Test

**Figure 3:**
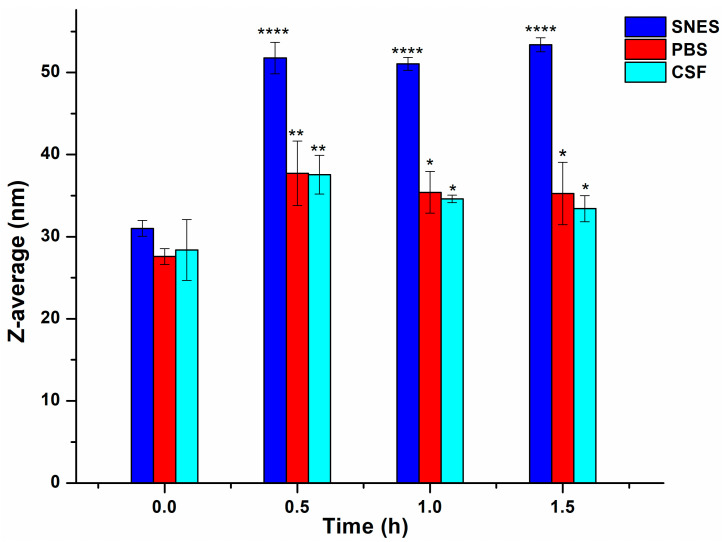
Micelle size changes (expressed as Z-average) in the presence of SNES (pH 5.6), PBS (pH 7.4), and CSF (pH 7.4). Results are presented as means ± SD (*n* = 3). Statistical analysis: *, *p* < 0.01; **, *p* < 0.001; ****, *p* < 0.00001. Statistical analysis is compared to the initial measured point.

The stability of optimized formulation in biological solutions, SNES, PBS, and CSF was evaluated by measuring the Z-average by the DLS method at predetermined time intervals. This test indicates the micelle’s behavior after intranasal administration. The obtained results in Figure 3 revealed a notable increase in Z-average after 0.5 h of incubation. Since biological media have a high ionic strength due to their elevated electrolyte content, it has been suggested in previous studies that they affect micelles in several aspects. Electrolytes with elevated concentrations might increase the physically entrapped water within the PEO chain network in the micelle corona, while disrupting the H-bonds between the water molecules and ether groups as a result of the dehydration and degradation of PEO chains. This collapse of PEO chains leads to a roughened micellar surface and greater asymmetry. Consequently, these effects contribute to an increase in the Z-average [62,63].

### 3.2. Physical Stability

**Figure 4:**
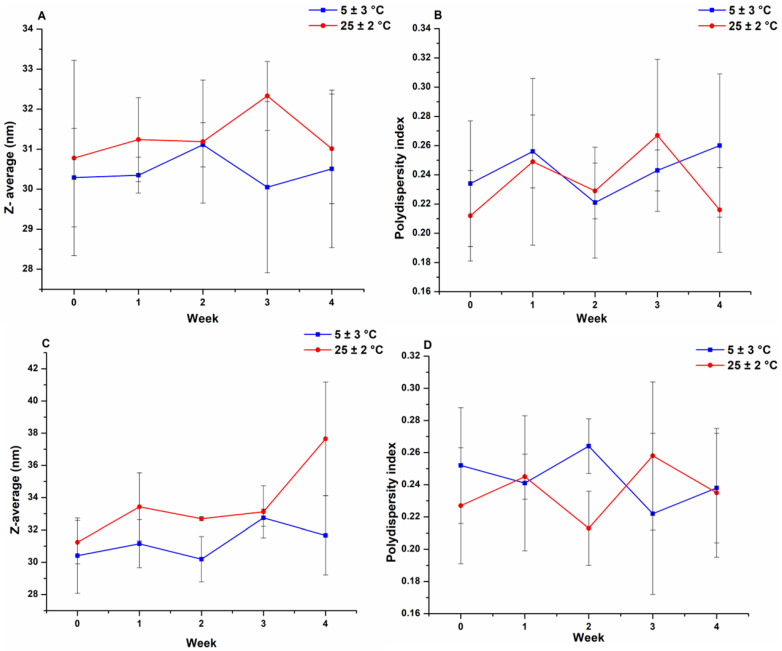
Results of physical stability investigation of CT-PM3: (**A**,**B**) solid state: Z-average (**A**) and PdI (**B**); (**C**,**D**) liquid state: Z-average (**C**) and PdI (**D**) at ambient temperature and fridge conditions. Results are presented as means ± SD (*n* = 3). All data are considered to be statistically non-significant (n.s., *p* > 0.01).

The results of the physical stability test can be found in Figure 4. There was only a slight increase in micelle size in the liquid state at 25 °C storage conditions, but it is statistically insignificant. Overall, the formulations show adequate stability and do not decrease polymeric micelle performance over the investigated period.

### 3.3. X-Ray Powder Diffraction Study (XRPD)

**Figure 5:**
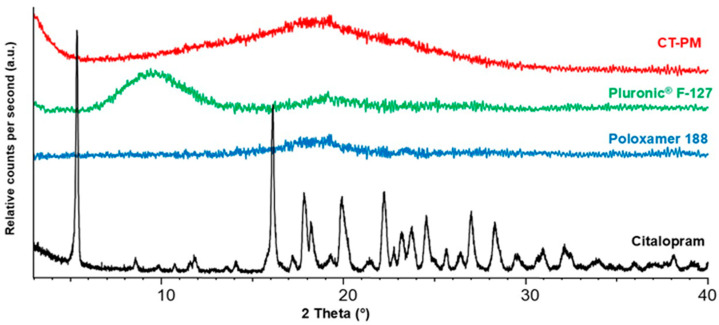
Diffractograms of CT-PM3, the initial polymeric micelle-forming components, and the active substance, citalopram (CT).

The X-ray powder diffraction study compared the crystallinity of the individual components and the final freeze-dried formulation (CT-PM3). The diffractograms can be seen in Figure 5. CT in its pure form indicates high crystallinity with sharp, intense peaks throughout the measured temperatures, especially between 5° and 30°. In comparison, the polymeric micelle-forming copolymers showed an amorphous nature. Poloxamer 188^®^ shows a broad halo pattern with no distinct sharp peak, and similarly, Pluronic F127^®^ shows a slightly more defined broad hump around 20°, which is typical of amorphous materials. The final formulation, CT-PM3, also shows an amorphous nature. It indicates complete amorphization due to the encapsulation of CT into the polymeric matrix, which in solid form acts as an amorphous molecular dispersion within the other amorphous polymers.

### 3.4. In Vitro Nasal Applicability Studies

#### 3.4.1. In Vitro Drug Release Study

**Figure 6:**
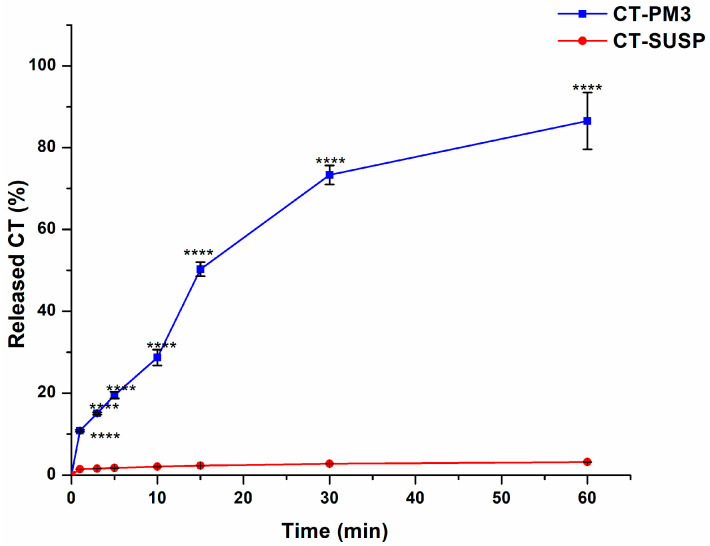
In vitro drug release profile of the optimized formulation in SNES (pH 5.6) compared to the initial CT. Results are presented as means ± SD (*n* = 3). Statistical analysis: ****, *p* < 0.00001.

Evaluating drug release is essential for assessing the efficiency of the drug delivery system compared to the initial drug, as it serves as a critical biopharmaceutical parameter. Based on the data in Figure 6, at each measured time point, CT-PM3 showed a significantly higher release rate (*p* < 0.0001) compared to the initial CT. The first 15–20 min is the crucial timeframe, as it is the duration of formulation residence in the nasal mucosa before being cleared by the mucociliary clearance mechanisms. [37]. At the 15 min mark, ca. 50% released from CT-PM3, which is 25 times higher than the ca. 2% release from pure CT. This accelerated drug release could be strongly associated with the reduction in micelle size and increased porosity as a result of the degradation of PEO chains in the micelle corona under the effect of temperature and electrolytes. [49,62,63]. Moreover, the obtained results emphasize the major increase in CT thermodynamic solubility.

Calculated parameters for drug release kinetics profiles are shown in Table 4. The highest value of R^2^ and the closest to 1.0 was considered as the criterion for choosing the fitted model [46]. The results revealed that the most appropriate kinetics model for initial CT release in SNES was the Korsmeyer–Peppas model (R^2^ = 0.9953). Although the Korsmeyer–Peppas model is commonly used to explain drug release from polymeric matrices, it has the flexibility to describe complex release situations with more than one mechanism in non-polymer-based systems [64,65], whereas the first-order model demonstrates the best fitted model of drug release from CT-PM3 (R^2^ = 0.9809), indicating the dependence of the release mechanism on the concentration of the drug within the micelles [66].

#### 3.4.2. In Vitro Drug Permeation Study

**Figure 7:**
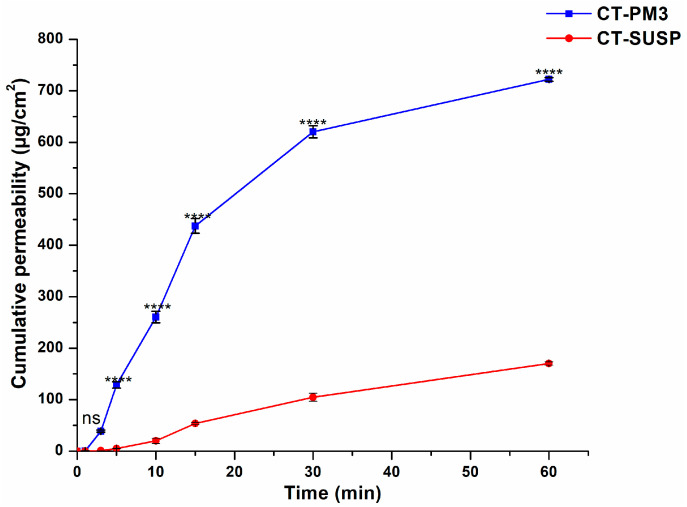
In vitro drug permeation study of the optimized formulation compared to the initial CT. Results are presented as means ± SD (*n* = 3). Statistical analysis: ****, *p* < 0.00001; ns, not significant.

**Figure 8:**
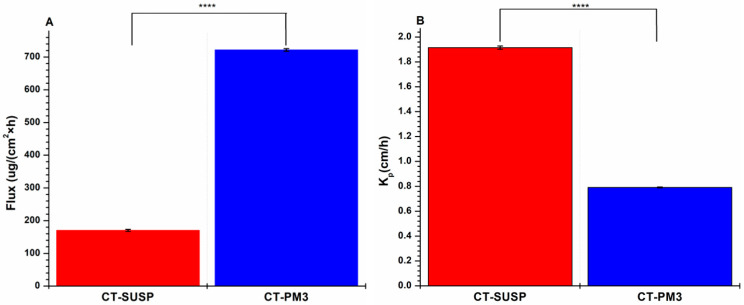
Calculated biopharmaceutical parameters of CT-PM3 cumulative permeability profile compared to initial CT (CT-SUSP). Flux (J) (**A**) and permeability coefficient (Kp) (**B**). Results are presented as means ± SD (*n* = 3). Statistical analysis: ****, *p* < 0.00001.

A modified Side-by-side^®^ horizontal diffusion test was performed to evaluate the in vitro nasal diffusion of CT-PM3 compared to the initial CT. The data, shown in Figure 7, revealed a significantly higher cumulative drug permeation for CT-PM3 at all measured intervals (*p* < 0.0001), with the optimized formulation achieving an approximately 4-fold increase over pure CT. In addition, the biopharmaceutical parameters (Figure 8) demonstrate the superior flux (J) of CT-PM3 compared to the initial CT.

This improvement is a result of the enhanced CT dissolution, micelle nanoscale size, micelle thermosensitive behavior, and the surfactant nature of the polymers, which collectively facilitate absorption through the membrane. The promoted permeability indicates the capability of this delivery system to provide a faster onset of action and enhance bioavailability via intranasal administration.

## 4. Discussion

In this study, a citalopram-loaded thermosensitive polymeric micelle formulation was developed and evaluated to assess its suitability as a brain-targeted intranasal delivery system for citalopram. This formulation was prepared by the thin-film hydration method, a widely used laboratory and pilot-scale technique for forming polymeric micelles and mixed micellar systems because it ensures good control over drug/polymer ratios, drug loading, and micelle size while remaining amenable to scale-up when process parameters are optimized [67]. Following formation, the product was stabilized and converted to a solid state; XRPD demonstrated an absence of crystalline reflections consistent with the drug residing in an amorphous state within the micellar structure [68]. Preservation of this amorphous character through solidification was achieved using freeze-drying. Several recent reports and reviews document that freeze-drying is an effective route to retain amorphous solid dispersion and to stabilize polymeric nanocarriers [69,70,71].

Physicochemical characterization at nasal temperature (35 °C) showed that the micelle size is approximately 31.4 nm with a PdI of 0.240, indicating a narrow size distribution and limited aggregation, as well as a size and homogeneity that favor diffusion across the nasal mucosa and reduced mucociliary clearance to larger particulates. Additionally, the near-neutral zeta potential enhanced mucosal compatibility and colloidal stability. Neutral or slightly negative surfaces often strike a balance between mucoadhesion and permeation for nose-to-brain targeting [72,73]. In stability testing, both liquid and freeze-dried forms maintained acceptable micelle size and colloidal performance after 4 weeks at ambient and refrigerated conditions, proving that freeze-drying was an effective strategy to ensure physical stability for this nanocarrier.

A notable functional attribute of the polymeric micelles was that it possesses a thermosensitive behavior, with an LCST value of approx. 31 °C. This value is kinetically convenient, as it supports stability at room temperature while facilitating a targeted triggered drug release at the nasal cavity temperature of 35 °C [32]. Blending Pluronics is also a highly reported tactic to tune gelation or thermosensitive nature, just like in this case for nasal applications.

The encapsulation efficiency of 88.85 ± 1.23% was achieved, signifying the quite efficient loading of CT into the micelle core. Furthermore, A 95-fold increase in CT thermodynamic solubility was achieved compared to the initial CT. The substantial 95-fold increase can be due the following synergistic mechanisms: (i) nanoscale solubilization with low micelle size; (ii) high encapsulation efficiency reducing the fraction of free crystalline drug; and (iii) the amorphous state of CT after freeze-drying, also contributing to rapid dissolution [74,75,76]. Incubation in biological media (SNES, PBS, and CSF) revealed a Z-average increase after 0.5 h due to ionic interactions and PEO chain deterioration. Nevertheless, the system demonstrated proper resistance to ionic strength fluctuations, confirming its suitability for intranasal delivery.

The in vitro drug release and permeation data further validated the formulation’s functional advantages, as a 25-fold increase in drug release and a 4-fold increase in nasal drug flux was experienced compared to unformulated CT. Such improvements are consistent with the effects of the highly efficient solubilization, the reduced diffusion path lengths for drug molecules desorbing from nanoscale carriers, and the thermokinetic behavior triggered by the nasal conditions [77,78].

## 5. Conclusions

In summary, it can be claimed that the thermosensitive polymeric micelle system is a promising approach to tackle the challenges of intranasal administration of brain-targeted drugs. However, further in vivo pharmacokinetics, long-term stability, and cytotoxicity investigations should be conducted to confirm safety, real efficacy, and reproducibility. Moreover, this formulation could serve as a base for additional developments in the field of intranasal delivery systems.

## Figures and Tables

**Table 1:** Composition of CT-loaded polymeric micelles formulations (CT-PM).

Formulation	CT (mg/mL)	P188 (mg)	PF127 (mg)
CT-PM1	5	40	90
CT-PM2	5	40	100
CT-PM3	5	40	110
CT-PM4	5	40	120

**Table 2:** Results of Z-average, PdI, and LCST measurements of CT-PM formulations. Data are presented as mean ± SD (*n* = 3).

Formulation	Z-Average at 35 °C (nm)	PdI at 35 °C	LCST (°C)
CT-PM1	36.44 ± 1.33	0.519 ± 0.44	29
CT-PM2	34.51 ± 2.47	0.447 ± 0.823	30
CT-PM3	31.41 ± 0.99	0.241 ± 0.029	31
CT-PM4	90.13 ± 0.23	0.365 ± 0.941	36

**Table 3:** Results of Z-average and PdI measurements of blank PM and CT-PM3. Data are presented as mean ± SD (*n* = 3).

Formulation	Z-Average at 35 °C (nm)	PdI
Blank PM	38.75 ± 3.24	0.248 ± 0.028
CT-PM3	31.41 ± 0.99	0.241 ± 0.029

**Table 4:** Obtained kinetic parameters of initial CT and CT-PM3.

Model		C-SUSP	CT-PM3
Zero-order	k_0_ (µg min^−1^)	0.071	1.767
	R^2^	−0.585	0.742
First-order	K_1_ (min^−1^) × 10^−3^	0.001	0.042
	R^2^	−0.564	0.981
Korsmeyer–Peppas	K_K-P_ (min^−n^) × 10^−3^	1.258	10.132
	*n*	0.225	0.539
	R^2^	0.995	0.964
Higuchi	k_H_ (µg min^−1/2^)	0.508	11.595
	R^2^	0.617	0.961
Hixon–Crowell	k_H-C_ (µg^1/3^ min^−1^) × 10^−3^	0.000	0.012
	R^2^	−0.571	0.964
Best fit		Korsmeyer–Peppas	First order

## Data Availability

Data are available upon request from the corresponding author.

## Abbreviations

The following abbreviations are used in this manuscript:

aCSF	artificially simulated cerebrospinal fluid
BBB	blood–brain barrier
CMC	critical micelle concentration
CT	citalopram
CT-PM	citalopram-loaded polymeric micelles
DLS	dynamic light scattering
EE	encapsulation efficiency
PF127	Pluronic F-127
FDA	food and drug administration
H-bonds	hydrogen bonds
HLB	hydrophilic–lipophilic balance
HPLC	high-performance liquid chromatography
IN	intranasal
IV	intravenous
LCST	low critical solution temperature
MPS	monophagocytic system
NCLs	nanostructured lipid carriers
P188	Poloxamer 188
PBS	phosphate buffer solution
PdI	polydispersity index
PEO	poly(ethylene oxide)
PES	polyether sulfone
PMs	polymeric micelles
PPO	poly(propylene oxide)
R^2^	regression coefficient
RES	reticuloendothelial system
SNES	simulated nasal electrolyte solution
SSRIs	selective serotonin reuptake inhibitors
XRPD	X-ray powder diffraction
WHO	World Health Organization
